# Whole-Genome Sequences of *Mycobacterium tuberculosis* TB282 and TB284, a Widespread and a Unique Strain, Respectively, Identified in a Previous Study of Tuberculosis Transmission in Central Los Angeles, California, USA

**DOI:** 10.1128/genomeA.01418-16

**Published:** 2017-01-12

**Authors:** Lixin Zhang, Zhenhua Yang

**Affiliations:** a Department of Epidemiology & Biostatistics, Michigan State University, East Lansing, Michigan, USA; b Department of Microbiology and Molecular Genetics, Michigan State University, East Lansing, Michigan, USA; c Department of Epidemiology, School of Public Health, University of Michigan, Ann Arbor, Michigan, USA

## Abstract

We report here the genome sequences of two *Mycobacterium tuberculosis* clinical isolates previously identified in central Los Angeles, CA, in the 1990s using a PacBio platform. Isolate TB282 represents a large-cluster strain that caused 27% of the tuberculosis cases, while TB284 represents a strain that caused disease in only one patient.

## GENOME ANNOUNCEMENT

Tuberculosis transmission has been associated with both patient- and environmental-related risk factors, such as the ability to generate infectious aerosols by the source patient, environmental conditions that affect the dispersal of aerosols, and susceptibility of exposed individuals (1). Molecular epidemiological studies have also shown that some *Mycobacterium tuberculosis* strains cause disease in many persons, whereas others only cause disease in a single or a limited number of patients (2–6). Why *M. tuberculosis* strains vary widely in the extent to which they cause disease in the population cannot always be explained by these epidemiological factors. In a previous study of tuberculosis transmission in central Los Angeles, CA, it was found that one *M. tuberculosis* strain, designated strain 210, was responsible for more than one-quarter of the tuberculosis cases (5). This large-cluster strain was found to grow more rapidly in human macrophages than strains that caused disease in a small number of persons or in a single patient who frequented the identified tuberculosis transmission sites when the patient was highly infectious (7). These findings suggest the potential importance of bacterial factors in tuberculosis transmission and pathogenesis. Here, we sequenced isolates TB282 and TB284 that represent strain 210 and the single-case-causing strain, respectively.

Genomic DNA was isolated using the cetyltrimethylammonium bromide (CTAB) method (8). Each genome was sequenced on a PacBio RSII platform with P5-C3 chemistry using one single-molecule real-time (SMRT) cell and a 10-kb insert library. *De novo* genome assemblies were created using PacBio’s SMRT Portal (version 2.2.0) and the Hierarchical Genome Assembly Process (HGAP) (9), with default settings and a seed read cutoff length of 5,000 bp. Annotation of the genomes was done using the RAST annotation server (10).

The single SMRT cell sequencing resulted in a total of 61,505 reads, with a mean read length of 7,292 bp (*N*_50_, 11,370 bp) and an average coverage of 82.95× for TB282, and a total 38,740 reads with a mean read length of 7,239 bp (*N*_50_, 11,083 bp) and an average coverage of 52.73× for TB284. Using filtered reads, a genome assembly with a single contig of 4,424,438 bp was obtained for TB282, and for TB284, an assembly with three contigs of 4,394,243 bp, 4,245 bp, and 1,737 bp, respectively, was obtained.

Both genome sequences have a mean G+C content of 65.6% and contain 45 tRNA genes and three rRNA genes. TB282 has 4,285 protein-coding regions, while TB284 has 4,230 protein-coding regions. The two genomes were compared by performing whole-genome alignment using Mauve (11) to identify sequence differences. A total of 2,147 single nucleotide polymorphisms (SNPs) were identified. In addition, there are 78 insertion or deletion differences greater than 100 bp that exist between the two genomes.

The genome sequences of this pair of isolates, one representing a widespread strain and the other a unique strain, will provide an additional resource for comparative genomic analyses in studying the bacterial factors important for tuberculosis transmission.

### Accession number(s).

The genome sequences of the *M. tuberculosis* strains TB282 and TB284 have been deposited at DDBJ/ENA/GenBank under GenBank accession numbers CP017920 and MNBY00000000, respectively.
